# Natural Is Not Always Safe: A Case of Torsades De Pointes Induced by Berberine Supplementation

**DOI:** 10.7759/cureus.111276

**Published:** 2026-06-22

**Authors:** Jeremy M Williams, Nikhita Yadlapalli, Francesco Franchi

**Affiliations:** 1 Internal Medicine, University of Florida (UF) Health, Jacksonville, USA; 2 Cardiology, University of Florida (UF) Health, Jacksonville, USA

**Keywords:** berberine, cardiac arrest, nutrition supplement, prolonged qtc interval, torsades de pointes (tdp)

## Abstract

Torsades de pointes (TdP) is a life-threatening form of polymorphic ventricular tachycardia (VT) that develops in the context of QT interval prolongation. Although many prescription medications are recognized as QT-prolonging agents, the arrhythmogenic risks associated with non-FDA-approved products, such as over-the-counter (OTC) and herbal supplements, are less understood. The widespread availability of these supplements online has led to increased use as holistic alternatives to conventional pharmacologic therapy. While certain supplements may offer potential benefits for chronic diseases, most remain non-FDA-approved and lack robust randomized clinical trials to assess their safety and adverse effect profiles. Here, we present the case of a 63-year-old woman who developed a prolonged QT interval of unknown origin and subsequently suffered cardiac arrest due to TdP. It was only immediately prior to a planned invasive procedure that the patient disclosed several months of berberine use, a herbal supplement purchased online. Following discontinuation of berberine, her symptoms and EKG abnormalities resolved completely. This case underscores the unknown and potentially fatal risks associated with OTC supplements promoted as holistic alternatives to medication. Increased patient education and physician awareness regarding the prevalence and dangers of supplement use are essential for ensuring patient safety and improving outcomes.

## Introduction

Torsades de pointes (TdP) is a distinctive form of polymorphic ventricular tachycardia (VT) characterized by a prolonged QT interval and a twisting morphology of QRS complexes around the isoelectric line on electrocardiography (ECG) [1]. It is most often associated with acquired or congenital abnormalities of cardiac repolarization and can rapidly progress to ventricular fibrillation, resulting in sudden cardiac death if not promptly recognized and treated [2]. Acquired QT prolongation is frequently associated with electrolyte imbalances, such as hypokalemia and hypomagnesemia, or is drug-induced. The latter has been reported with a wide range of medications, including antiarrhythmics, antimicrobials, psychotropic agents, and other commonly prescribed therapies [3,4]. Importantly, the increasing use of over-the-counter (OTC) supplements introduces additional, often underrecognized sources of QT-prolonging exposure.

​The pathophysiology of TdP centers on delayed and uneven repolarization in cardiac myocytes, as evidenced on ECG by QT interval prolongation [5]. This occurs due to either reduced outward potassium (K+) currents and/or persistent inward sodium (Na+)/calcium (Ca2+) currents across the cell membrane, resulting in a prolonged action potential duration [5,6]. When the degree of prolongation does not affect all cells uniformly, some cells remain in the refractory period longer than others, resulting in a spatial dispersion of repolarization. Additionally, if the action potential duration is abnormally prolonged, voltage-gated Ca2+ channels can prematurely reactivate, leading to a secondary depolarization phase known as early afterdepolarizations (EADs) [7,8]. These EADs can reach threshold and subsequently trigger a premature ventricular contraction (PVC). The combination of spatial dispersion of repolarization and the occurrence of EADs creates zones of excitable tissue next to refractory tissue, favoring reentry or shifting of focal activity once a PVC occurs [4-6]. The interaction of the triggered PVC with this unstable substrate leads to a polymorphic VT whose QRS axis appears to 'twist' around the baseline. Most episodes self‑terminate when dispersion of repolarization diminishes, but some progress to ventricular fibrillation and potentially sudden cardiac death [4,9,10].

​Berberine is a plant‐derived alkaloid commonly used in traditional Chinese medicine, usually for gastrointestinal upset. It is now marketed as a natural remedy for a wide range of conditions, including metabolic disorders, cardiovascular disease, infections, cancer, and various inflammatory and neurodegenerative conditions [11]. While some randomized controlled trials in humans support moderate improvements in blood glucose, insulin resistance, lipid profiles, and some inflammatory markers, the overall evidence base and trial quality are variable, with many indications still lacking large, long-term studies [11,12]. Furthermore, most sales of the product lack FDA approval and thus harbor potential for serious and potentially life-threatening side effects. Mechanistically, berberine affects multiple signaling pathways throughout the body and modulates ion channels at the cell membrane. In relation to this case, berberine notably interacts with key potassium channels on the cardiac membrane; specifically, berberine binds to and inhibits delayed rectifier K+ currents such as hERG and IKr. Blockade of these channels results in diminished outward K+ current, which ultimately leads to prolonged action potential duration, delayed ventricular repolarization, and potentially TdP [12-15].

While physicians are aware of the established QT-prolonging agents, less is known about the arrhythmogenic potential of commonly used OTC supplements. Current literature describes only three other case reports specifically noting berberine-induced TdP. This case adds to the small but growing number of reports of berberine-associated TdP and highlights the need for greater awareness among clinicians of its potential to cause clinically significant QT prolongation and life-threatening ventricular arrhythmias.

## Case presentation

A 63-year-old woman with a history of hypertension, type 2 diabetes mellitus, and hyperlipidemia presented to the emergency department after experiencing a syncopal episode. She described being in her usual state of health while grocery shopping, but upon returning home, she felt a brief fluttering sensation in her chest followed by a complete loss of consciousness lasting approximately 10 seconds. The episode was witnessed by family members, who did not observe any seizure-like activity. Upon awakening, she reported no post-ictal confusion and denied any prior syncopal events, chest pain, shortness of breath, or a family history of prolonged QT. Her home medications included amlodipine 5 mg daily, losartan 50 mg daily, and metformin 1000 mg twice daily.

**Figure 1:**
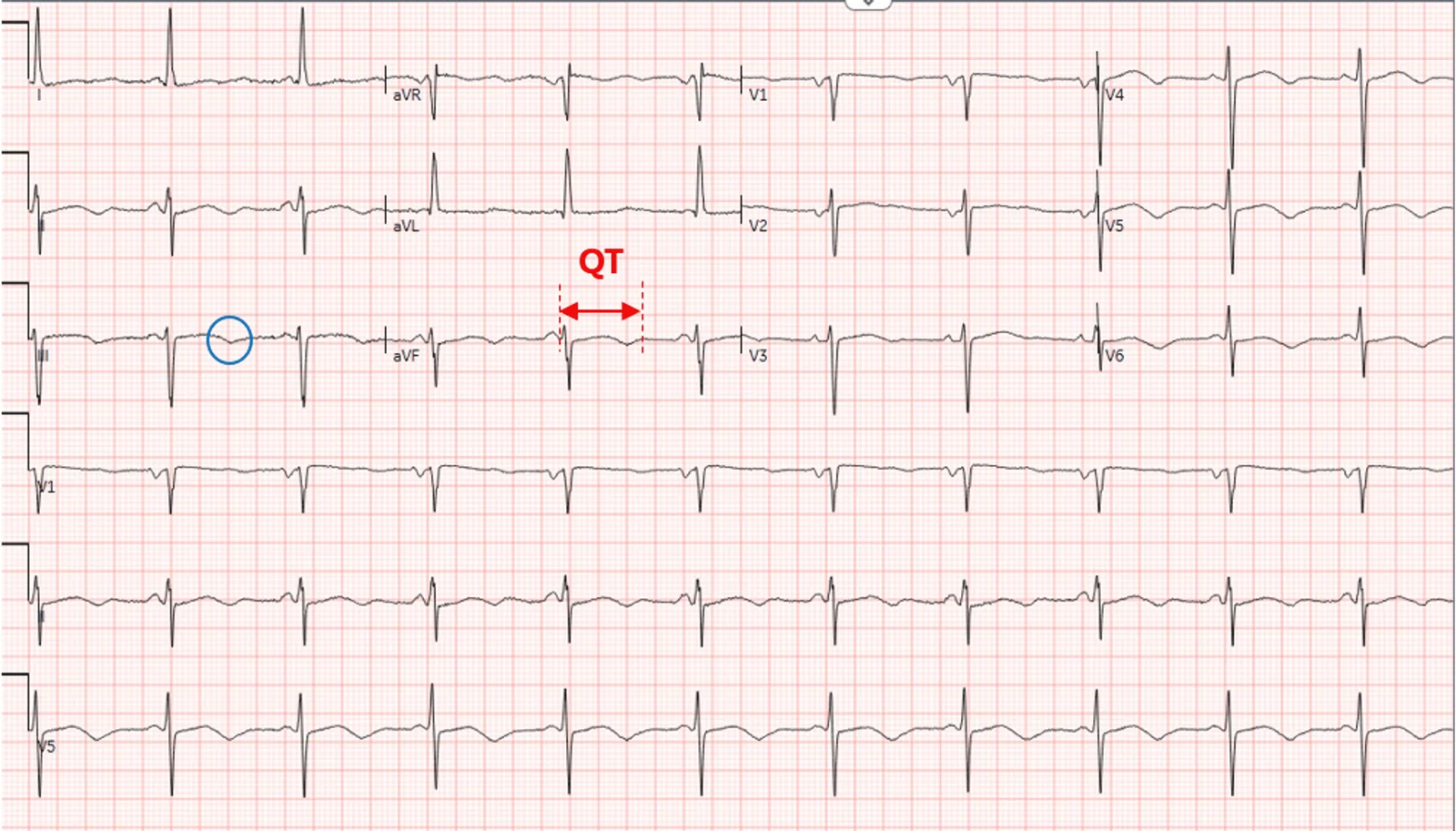
The ECG on admission showed a normal sinus rhythm, diffuse T-wave inversions (blue circle in lead III), and a prolonged QTc of 572 ms (red double-sided arrow in lead augmented vector foot (aVF)) ECG: Electrocardiogram, aVF: Augmented vector foot

​Upon arrival, her blood pressure was elevated at 176/84 mmHg, while heart rate, temperature, and oxygen saturation were within normal limits. Physical examination was notable for diaphoresis but was otherwise unremarkable. Laboratory tests showed mild elevations in high-sensitivity troponin levels (25, 34, and 44), with all other results, including potassium and magnesium levels, found to be within normal ranges. An ECG revealed normal sinus rhythm, diffuse T-wave inversions, and a prolonged QTc of 572 ms, with no significant ST elevation or depression to suggest acute ischemia (Figure 1).

**Figure 2:**
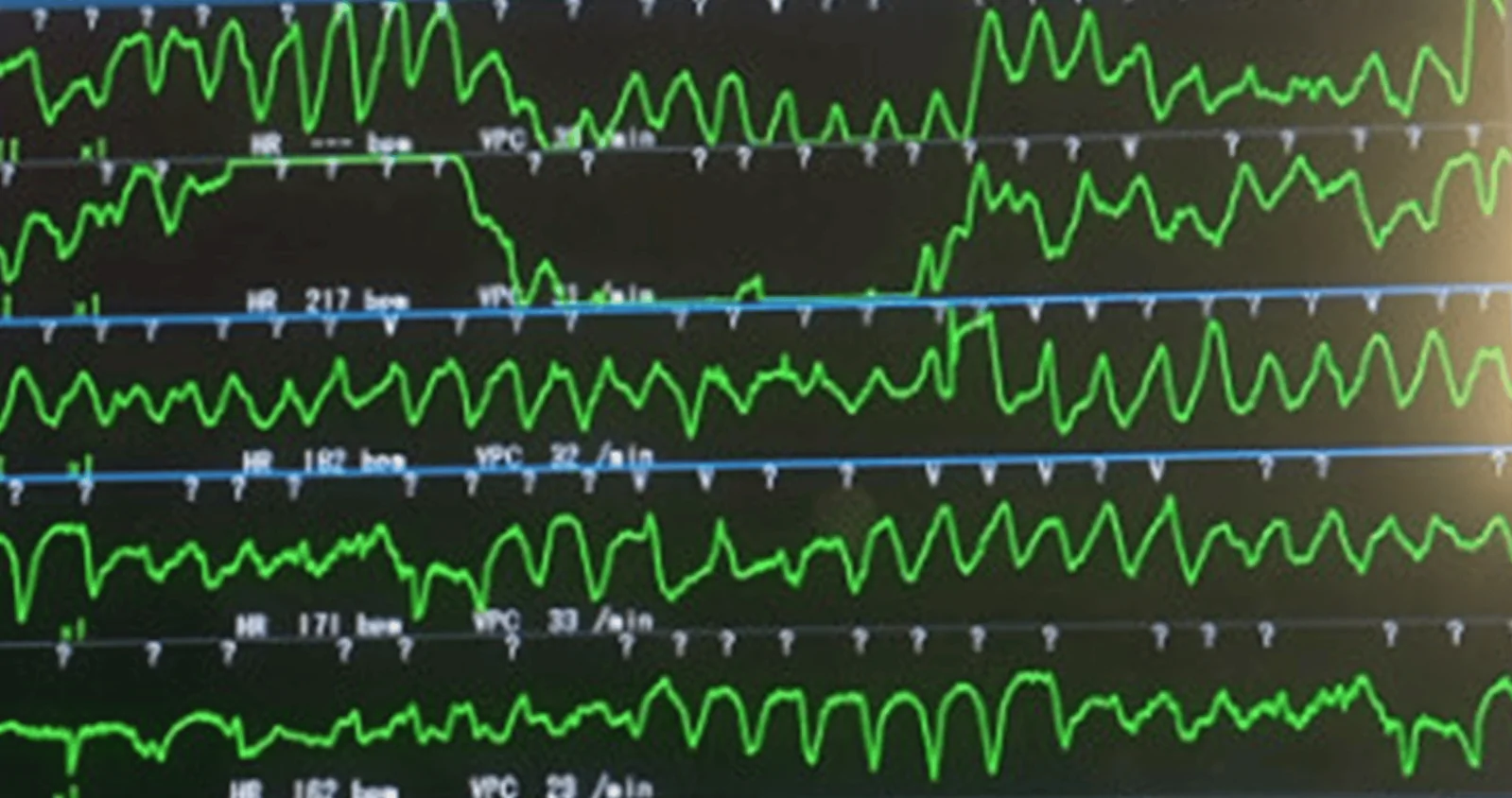
Telemetry monitoring showed a polymorphic wide-complex tachycardia consistent with TdP TdP: Torsades de pointes

While in the emergency department, the patient became acutely unresponsive. Telemetry monitoring revealed a polymorphic wide-complex tachycardia consistent with TdP (Figure 2). She quickly regained consciousness and spontaneously returned to normal sinus rhythm without the need for acute intervention. For cardiac membrane stabilization, she received 4 g of intravenous calcium gluconate and 4 g of intravenous magnesium and was admitted to the cardiac intensive care unit for further monitoring. Left heart catheterization demonstrated mild nonobstructive coronary artery disease, and medical management was recommended with aspirin 81 mg daily and rosuvastatin 20 mg daily. Transthoracic echocardiography revealed a left ventricular ejection fraction of 50% to 55%, mild left ventricular hypertrophy, mild to moderate aortic regurgitation, and moderate mitral regurgitation. Cardiac MRI showed no evidence of acute or chronic infarction, fibrosis, or myocarditis. The electrophysiology team planned for implantable cardioverter-defibrillator (ICD) placement as secondary prevention. However, further questioning before the procedure revealed the patient had been taking OTC supplements, including tauroursodeoxycholic acid (TUDCA) for liver health and berberine for diabetes management. Specifically, the patient reported taking berberine 450 mg twice daily for six months prior to presentation. None of the patient's prescribed home medications or TUDCA has a known QT-prolonging effect, making berberine the likely culprit. Since this discovery represented a reversible cause, ICD placement was canceled. The patient was monitored for an additional 48 hours with no recurrence of syncope or ventricular arrhythmias. A repeat ECG prior to discharge showed normalization of the QT interval to 436 ms. She received education on avoiding QT-prolonging medications and was discharged with plans for outpatient electrophysiology follow-up in two weeks.

## Discussion

Torsades de pointes is a form of polymorphic VT that most commonly arises in the context of prolonged QT syndrome. This arrhythmia may resolve spontaneously or progress to ventricular fibrillation, resulting in cardiac arrest. Prolonged QT syndrome can be broadly categorized as either congenital, due to genetic mutations in ion channels, or acquired, such as from QT-prolonging drugs or electrolyte disturbances. In clinical practice, drug-induced QT prolongation is more frequently encountered, with well-known causative agents including antiarrhythmics, antimicrobials, and psychotropic drugs [1-5]. Although clinicians are typically vigilant when prescribing medications known to prolong the QT interval, the increasing use of OTC supplements has introduced additional, often unrecognized, sources of QT-prolonging exposure. Although the incidence of TdP resulting from drug-induced QT prolongation is relatively low, approximately 0.3%, it is essential for practitioners to recognize underreported causes such as OTC supplements like berberine, given their potential to precipitate life-threatening arrhythmias [16].

​This case contributes to the limited clinical literature on berberine-associated TdP, with only a few published reports to date. In these cases, patients typically lacked traditional TdP risk factors aside from female sex and occasional electrolyte imbalances. Notably, arrhythmias resolved following berberine withdrawal, suggesting the supplement may act as a primary trigger rather than a minor contributor [15,16]. Using the Naranjo scale, this case scores as 'probable' adverse drug reaction (score 6-7) due to a positive challenge and absence of alternative explanations. Unlike many prescription QT-prolonging agents, berberine is marketed as a 'natural' product, often perceived as inherently safe, and is commonly used without medical supervision or documentation in medication lists. Such under-recognition can result in under-reporting of adverse events and underestimation of the true arrhythmogenic risk.

​The clinical course in our patient emphasizes a key management principle for TdP; while an implantable cardioverter-defibrillator (ICD) is generally recommended for secondary prevention in patients at high risk of recurrence, such as those with congenital long QT syndrome, it is not indicated when the underlying cause is reversible. Although the patient had mild structural heart disease noted on a transthoracic echocardiogram, the absence of persistent QT prolongation after berberine discontinuation and the lack of recurrent arrhythmias supported deferring ICD placement, consistent with guidelines that prioritize reversible cause correction [17]. In this case, identifying berberine as a reversible etiology prevented unnecessary interventions such as ICD placement. This underscores the importance of thoroughly reviewing all substances ingested by the patient, including prescription medications, OTC drugs, and especially supplements, when evaluating unexplained syncope, QT prolongation, or polymorphic VT. Failure to identify supplement use can result in recurrent arrhythmias and potentially unwarranted invasive procedures.

​This report highlights several important clinical considerations. Clinicians should routinely ask about berberine and other supplement use when assessing patients with syncope or abnormal ECG findings. Patient education regarding the risks of QT-prolonging supplements, especially berberine, is critical for individuals with baseline QT prolongation or those already taking known QT-prolonging medications. If berberine use is identified in the context of QT prolongation, immediate discontinuation and close QT interval monitoring are essential for patient safety. Enhancing awareness and early recognition of berberine as a precipitating factor in cases of new QT prolongation or TdP can prevent unnecessary invasive procedures and ultimately improve clinical outcomes.

## Conclusions

This case underscores the potentially life-threatening arrhythmogenic risks associated with berberine, a widely used OTC herbal supplement promoted as a 'natural' remedy for metabolic and cardiovascular conditions. In our patient, unexplained QT prolongation escalated to TdP and cardiac arrest, with both clinical and electrocardiographic abnormalities resolving completely after discontinuation of berberine. As the use of non-FDA-regulated supplements increases, clinicians must maintain a high index of suspicion for supplement-induced QT prolongation when assessing patients with syncope, ventricular arrhythmias, or unexplained repolarization abnormalities. Obtaining a comprehensive medication history, including explicit questions about herbal and OTC products, is vital, as overlooking these agents may lead to recurrent arrhythmias, unnecessary invasive procedures, and avoidable morbidity or mortality. Clinicians should specifically ask about berberine and consider it a potential cause of unexplained QT prolongation or TdP. Enhanced physician awareness, patient education, and further research into the cardiovascular safety of commonly used supplements, such as berberine, are essential to improving patient outcomes.
