# Correction: Natural Is Not Always Safe: A Case of Torsades De Pointes Induced by Berberine Supplementation

**DOI:** 10.7759/cureus.c476

**Published:** 2026-09-03

**Authors:** Jeremy M Williams, Nikhita Yadlapalli, Francesco Franchi

**Affiliations:** 1 Internal Medicine, University of Florida (UF) Health, Jacksonville, USA; 2 Cardiology, University of Florida (UF) Health, Jacksonville, USA

This article has been updated to change the patient’s age to 63. Previously this was misstated as 36.

